# The Impact of Virtual-Reality-Based Physiotherapy on Upper Limb Function in Children with Cerebral Palsy

**DOI:** 10.3390/jcm14197040

**Published:** 2025-10-05

**Authors:** Zuzanna Wojtowicz, Katarzyna Czech, Adrianna Lechowska, Justyna Paprocka

**Affiliations:** 1 Doctoral School, Medical University of Silesia in Katowice, 40-055 Katowice, Poland; 2 Faculty of Medical Sciences in Katowice, Medical University of Silesia in Katowice, 40-055 Katowice, Poland; 3 Pediatric Neurology Department, Faculty of Medical Sciences, Medical University of Silesia in Katowice, 40-752 Katowice, Poland

**Keywords:** cerebral palsy, virtual reality, upper limb

## Abstract

**Background/Objectives**: Cerebral palsy (CP) is one of the most common causes of permanent motor disability in children, and its consequences for upper limb function have a significant impact on the patient’s independence and quality of life. Virtual reality is attracting increasing interest as a modern, engaging and effective method of physiotherapy for children with cerebral palsy. This systematic literature review aimed to synthesize current scientific data on the impact of virtual-reality-based physiotherapy on upper limb function in children with cerebral palsy. **Methods**: The review was conducted in accordance with PRISMA 2020 guidelines. PubMed, Science Direct, Scopus, Web of Science, Research Gate and Google Scholar databases were searched for studies published between 2010 and 2025. Six original studies meeting the following criteria were included in the analysis: virtual reality therapy, population of children with cerebral palsy, physiotherapy goals related to the upper limb and availability of full text. **Results**: All included studies demonstrated a positive impact of virtual reality on at least one functional parameter of the upper limb, including range of motion, muscle strength, coordination and manual precision. Task-oriented training, immersive virtual reality environments and home-based therapy supported by remote monitoring proved to be the most effective. The effects were particularly noticeable in children with moderate impairment at GMFCS I–III. **Conclusions**: Virtual reality represents a safe and promising technology to support upper limb physiotherapy in children with cerebral palsy. It can be used both in clinical and home settings, contributing to increased exercise intensity and motivation. Further long-term studies using high-quality methodology are needed to determine the sustainability of the effects and their impact on everyday living.

## 1. Introduction

Cerebral palsy (CP) is one of the most common non-progressive disorders of the central nervous system in children, leading to irreversible motor and postural disorders and often accompanied by cognitive, sensory and perceptual deficits [1]. Upper limb dysfunction is one of the most difficult clinical aspects of cerebral palsy to improve, particularly in hemiplegic and tetraplegic patients. Impaired mobility, precision of movements and coordination of the upper limb significantly reduce the child’s independence and have a significant impact on the quality of life of the entire family [2].

In recent years, new neurorehabilitation methods based on advanced technology, including virtual reality (VR) systems, have been developed and refined. A VR system is defined as a computer-generated three-dimensional interactive environment that can simulate sensory and motor stimuli, allowing the patient to participate in therapy actively [3]. This technology finds application in various areas of physiotherapy, and its adaptation to hand therapy in children with cerebral palsy opens up new possibilities for neuroplasticity and personalization of exercises during therapy [4].

Virtual reality enables the repeated performance of targeted motor activities in an environment that engages the patient emotionally and cognitively, which may promote the activation of brain reorganization mechanisms [5]. Research suggests that virtual reality may be an effective therapeutic tool to support traditional therapies, offering additional benefits such as increased patient motivation, greater engagement and better treatment compliance [6].

Despite the growing number of publications on this subject, the results of previous studies are inconclusive, and many show methodological limitations. Therefore, this article aims to review current scientific studies on the effectiveness of physiotherapy using virtual reality in improving upper limb function in children with cerebral palsy. This review was prepared in accordance with the PRISMA 2020 guidelines, which ensure a high standard of reliability and transparency in reporting. A key element of the definition of CP is that the primary brain damage does not worsen over time, but its clinical manifestations may evolve as the child’s nervous system matures [7].

According to the accepted classification published by the International Committee for the Definition and Classification of CP (2006), CP describes movement and postural disorders that lead to activity limitations and are attributed to non-progressive damage to the developing brain [8]. These disorders often coexist with other neurological deficits, such as cognitive, sensory, communication and behavioral impairments, and epilepsy [9].

The prevalence of CP in the general population ranges from 1.5 to 3 cases per 1000 live births and remains relatively stable despite advances in perinatology and neonatology [10]. Its prevalence is higher among premature infants and those with very low birth weight, highlighting the key risk factor of central nervous system immaturity [11]. It should be emphasized that CP is not a disease entity in the classical sense—rather, it is a spectrum of symptoms resulting from brain damage of various etiologies. Therefore, in clinical practice, it is crucial not only to establish a general diagnosis but also to determine the type of disorder (e.g., spastic, ataxic, dyskinetic), its topography and functional severity [12].

The definition of CP also encompasses the variability of symptoms over time, determined by the maturation of the nervous system and the impact of therapy. Children with this diagnosis may demonstrate improvement in motor function following appropriate therapeutic interventions, but the brain damage remains irreversible [13].

The multidimensional nature of symptoms makes CP one of the most complex neurological disorders of development, requiring an interdisciplinary approach. This complexity translates into the need for individualized diagnosis and treatment, which must be tailored not only to the child’s age but also to the functional level and socio-familial context [14].

## 2. Materials and Methods

This literature review was conducted according to the PRISMA (Preferred Reporting Items for Systematic Reviews and Meta-Analyses) 2020 guidelines to ensure reliability, transparency and a high methodological standard. The study stages included literature review, study selection, definition of inclusion and exclusion criteria, data analysis, and summary of results.

### 2.1. Databases and Search Strategy

A systematic literature search was conducted using the following electronic databases:

- PubMed/MEDLINE;
- ScienceDirect;
- Scopus;
- Web of Science;
- ResearchGate;
- Google Scholar.

The search covered papers published between January 2010 and June 2025. The following combination of keywords and logical operators was used:

- “cerebral palsy” or “spastic hemiplegia”;
- “virtual reality” or “VR therapy”;
- “upper limb” or “upper extremity” or “hand function”;
- “rehabilitation” or “physiotherapy”;
- “children” or “pediatrics”.

In addition, a manual search of the bibliography in primary research articles and systematic reviews was conducted.

### 2.2. Inclusion Criteria

Studies meeting the following conditions were included in the review:

- Availability of full text;
- Language of publication: Polish or English;
- Population: children under 18 with clinically diagnosed cerebral palsy (any type);
- Intervention: physiotherapy/rehabilitation using virtual reality;
- Therapy goal: improvement of upper limb or hand function—muscle strength, range of movement, coordination;
- Type of study: randomized controlled trials (RCTs), quasi-experimental or prospective studies with a control group.

### 2.3. Exclusion Criteria

The following were excluded from the review:

- Studies involving only adults;
- Studies involving only the lower limb;
- Studies without the use of virtual reality, e.g., robotics, classic physiotherapy;
- Single case reports or case series;
- Expert opinions without quantitative data and narrative reviews.

### 2.4. Selection Process

**Figure 1:**
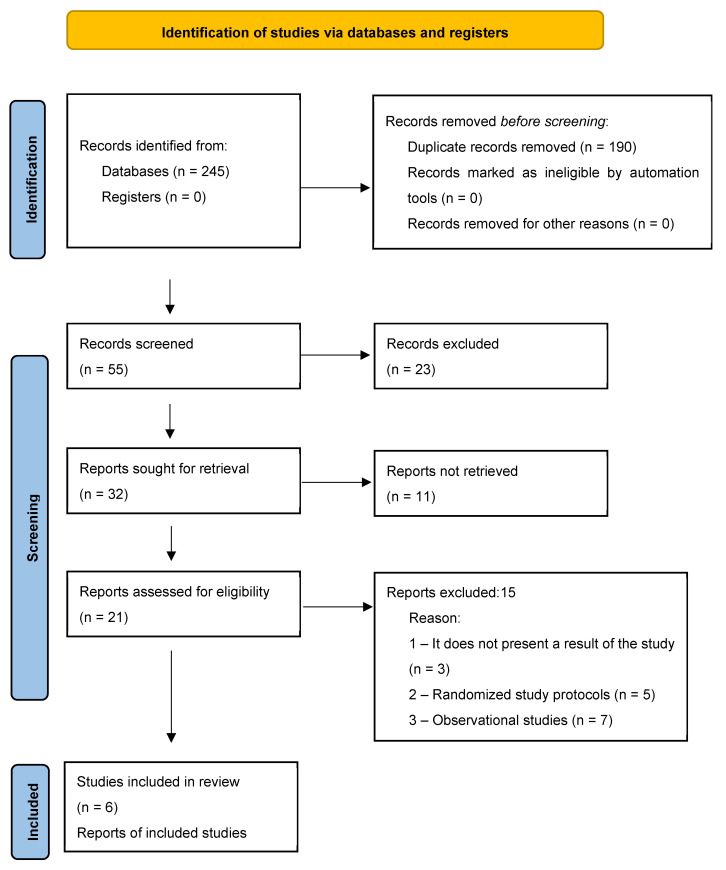
PRISMA 2020 flow diagram for new systematic reviews which included searches of databases and registers only. PRISMA, Preferred Reporting Items for Systematic Reviews and Meta--Analyses.

Two independent sources reviewed the titles and abstracts to ensure they met the inclusion criteria. The full texts of the selected papers were then evaluated to make the final selection. Any disagreements were resolved by consensus. The PRISMA 2020 flow diagram was used to illustrate the selection process (Figure 1).

### 2.5. Data Extraction and Analysis

Data from the included studies are summarized in Table 1. They include: author and year of publication, study type, number of participants, type of virtual reality intervention, assessment tools and main results. No meta-analysis was performed due to considerable methodological heterogeneity (different types of virtual reality, duration of intervention).

### 2.6. Quality Assessment and Risk Bias

The methodological quality and risk of bias of the nine studies included in this review were evaluated using the Cochrane Risk of Bias 2.0 tool for randomized controlled trials. The following domains were analyzed: the randomization process, allocation concealment, blinding of participants and personnel, completeness of outcome data, selective reporting and other potential sources of bias. Two reviewers independently assessed each study, and any discrepancies were resolved through discussion.

#### 2.6.1. Assessment Results

- Randomization and allocation concealment: In all studies, the randomization process was described; however, detailed information regarding the generation of the random sequence and allocation concealment was incomplete, resulting in a judgment of moderate risk of bias.
- Blinding: None of the studies implemented full blinding of participants or therapists, which increases the risk of placebo effects and subjective outcome assessment (moderate to high risk of bias).
- Completeness of data: Most studies reported complete outcome data; however, due to the small sample sizes (10–41 participants), even minimal dropout could have influenced the results (moderate risk of bias).
- Selective reporting: No clear evidence of selective reporting was identified; nevertheless, the absence of preregistered protocols limited the certainty of this assessment (low to moderate risk of bias).
- Other sources of bias: The main limitation was the heterogeneity of interventions (different VR systems, duration and frequency of sessions), which hinders comparability of results and may introduce confounding.

#### 2.6.2. Summary

Overall, the methodological quality of the included studies was judged as moderate. The most frequent sources of bias were the lack of blinding and the small sample sizes, both of which limited the statistical power of the analyses. Despite these limitations, all studies consistently reported beneficial effects of VR-based physiotherapy on upper limb function in children with cerebral palsy, which strengthens the reliability of the findings.

## 3. Results

As a result of database searches and manual selection of bibliographies, 245 records were identified. After removing duplicates and reviewing abstracts, 21 papers were selected for full-text evaluation. The final selection of nine original studies was made according to the criteria for inclusion in this literature review. The PRISMA 2020 flow diagram is shown in Table 1.

### 3.1. Characteristics of the Included Studies

All included studies were prospective, of which five were randomized controlled trials (RCTs) and one was a meta-analysis of 14 primary research experiments. The studies came from various research centers (Europe, North America, Asia) and included children with cerebral palsy, mainly hemiplegia or diplegia.

The number of participants ranged from 10 to 41 in a single study, with the largest study involving 41 children with cerebral palsy aged 4–18 years [18]. The interventions involved the use of virtual reality with varying degrees of immersion and functional adaptation, from commercial systems to specialized platforms designed for hand therapy, e.g., IMU, Immertrack and Leap Motion devices.

### 3.2. Type of Interventions

The study included a variety of therapeutic protocols:

- Classic VR therapeutic protocols with interactive biofeedback [24];
- VR task-oriented training with fine motor elements [25];
- Gamified VR with motivational educational apps [26];
- Immersive VR therapy based on 3D environments with motion control [27];
- In-home VR therapy supported by sensors and remote monitoring [28].

The interventions lasted from four to eight weeks, most often with three sessions per week of 30–45 min.

### 3.3. Assessment Tools and Results

The most common assessment tools included the following:

- Assisting Hand Assessment (AHA);
- Quality of Upper Extremity Skills Test (QUEST);
- ABILHAND-Kids;
- Melbourne Assessment 2 (MA2);
- Biomechanical measurements: ROM, grip strength, task time.

All studies reported improvement in at least one upper limb function parameter (range of motion, muscle strength, precision, manual functionality) (Table 2). The greatest benefits were observed in the following areas:

- Improved bilateral coordination;
- Increased movement precision;
- Reduced time to perform functional tasks [29,30,31,32,33].

**Table 2:** Assessment tools and detailed results of VR therapy in children with cerebral palsy.

	Authors (Year)	Assessment Tools	Results and Effects
1	Menekseoglu et al. (2023) [15]	Assisting Hand Assessment (AHA), ABILHAND-Kids, Quality of Upper Extremity Skills Test (QUEST), KINDL and active range of motion.	- Improved two-handed activities; - Subjective ease of task performance; - Increased range of motion in flexion, pronation and supination of the hand; - Subjective feeling of improvement in hand dexterity.
2	Choi et al. (2023) [16]	Melbourne Assessment of Unilateral Upper Limb Function v2 (MA2), ULPRS, PEDI-CAT, 3D movement analysis, satisfaction questionnaires.	- Improved movement precision; - Improved segmental control; - Improved daily activities and social and cognitive functions; - Reduced movement time, improved shoulder movement pattern based on 3D analysis.
3	Goyal et al. (2022) [17]	ABILHAND-Kids, WeeFIM, 9HPT, BBT	- Improved movement precision; - Improved daily activities and social and cognitive functions; - Improved motor control and muscle strength.
4	Metin Ökmen et al. (2019) [18]	Bimanual Fine Motor Function (BFMF), FMS	- Improved segmental control; - Improved motor control and muscle strength.
5	Daliri et al. (2025) [19]	Spoon use time—daily activity, knife use time—daily activity, range of motion (ROM) and grip strength.	- Increased precision and speed of hand movements during daily activities; - Improved motor control and muscle strength; - No improvement in wrist extension.
6	Saussez et al. (2023) [20]	Assisting Hand Assessment (AHA), BBT-MA, BBT-LA, JTTHF-MA hand, JTTHF-LA hand, MFPT-MA hand, MFPT-LA hand, 6MWT, ABILHAND-Kids, ACTIVLIM-CP, PEDI, ABILOCO-Kids, COPM perf, COPM sat	- Improved two-handed activities; - Subjective ease of task performance; - Increased range of motion in flexion, pronation and supination of the hand; - Subjective feeling of improvement in hand dexterity.
7	Baillet et al. (2024) [21]	Movement Assessment Battery for Children 2 (MABC-2), range of motion (ROM) assessment,	- Improved range of motion; - Improved motivation; - Increased movement precision; - Increased movement fluidity.
8	Avcil et al. (2021) [22]	Minnesota Manual Dexterity Test (MMDT), Childhood Health Assessment Questionnaire (CHAQ), Duruoz Hand Index (DHI), dynamometer.	- Increased manual dexterity; - Increased grip and squeeze strength; - Improved hand function.
9	Golomb et al. (2010) [23]	Assessment of finger range of motion (ROM) using a sensor glove, dual-energy X-ray absorptiometry (DXA), peripheral quantitative computed tomography (pQCT) and functional magnetic resonance imaging (MRI).	- Improvement in activities of daily living; - Increased range of motion of fingers as measured by the glove. - In two participants with better treatment compliance, an improvement in the mineral content of the radius and its surface area in the affected limb was observed; - fMRI showed increased activation in motor networks.

### 3.4. Heterogeneity and Limitations

The study results were positive but showed considerable heterogeneity in terms of methodology: different types of VR, treatment protocols that deviated in terms of duration, outcome measures and a small number of participants. A meta-analysis by Chen et al. [14] indicated a large overall effect of VR therapy (d = 1.00) but highlighted the poor methodological quality of some of the primary studies.

## 4. Discussion

The findings of this literature review illustrate the growing interest within the scientific and clinical community in the use of virtual reality (VR) in upper limb physiotherapy for children with cerebral palsy (CP). VR is increasingly being investigated not only as an adjunct to conventional therapies, but also as a potential form of stand-alone or hybrid intervention, including remote and home-based applications. Evidence from the six randomized controlled trials included in this review suggests that VR interventions may be associated with improvements in functional, motor and coordination outcomes in children with various types of cerebral palsy, particularly spastic hemiplegia.

Menekseoglu et al. (2023) [15] evaluated gamified VR physiotherapy and reported statistically significant improvements in upper limb function, observed both in quantitative measures (e.g., range of motion) and qualitative assessments (e.g., AHA, QUEST). These effects were also maintained at follow-up, which may indicate the possibility of longer-term impacts of VR on neuroplastic processes. VR interventions have been proposed to activate multiple cortical regions responsible for motor planning, visuomotor perception, and sensory integration, which could help explain the observed outcomes [34].

In contrast, home-based studies, such as that by Choi et al. (2023) [16], highlight the potential of VR delivered through accessible mobile technologies (IMU sensors) and remote monitoring. Such approaches may be particularly relevant in contexts of limited access to rehabilitation services, as well as during situations such as the COVID-19 pandemic. The systematic application of VR in home environments could contribute to reducing disparities in access to therapy and may support greater family involvement in the child’s exercise management.

Another notable aspect of the reviewed studies is the technological diversity of VR systems. These ranged from immersive platforms (e.g., Immertrack), to devices capable of capturing detailed limb movements (e.g., Leap Motion), to semi-commercial solutions and educational applications. Daliri et al. (2025) [19] investigated task-oriented VR therapy (VR-TOT) and observed improvements in grip strength and in the time required to complete daily activities, such as eating with a spoon. This type of task-oriented training aligns with contemporary principles of neurorehabilitation, which emphasize active, functional and varied exercises performed in contextually relevant settings.

It has also been suggested that virtual reality may provide emotional and motivational benefits. In contrast to traditional rehabilitation exercises, which many children perceive as monotonous and repetitive, VR environments can present visually engaging scenarios that are more likely to sustain concentration and maintain attention. Increased motivation has been proposed as one of the factors that may influence the outcomes of VR therapy, particularly in children who find it difficult to remain focused in conventional therapy settings [35].

From a neurophysiological perspective, the potential effectiveness of VR in children with cerebral palsy has been linked to the theory of neuroplasticity. Intensive and multisensory training may encourage reorganization of the nervous system through the formation of new synaptic connections and the activation of alternative neural pathways. Stimuli generated in VR—visual, auditory and proprioceptive—can simultaneously stimulate multiple brain regions, including the motor cortex, prefrontal cortex, cerebellum and limbic system [36]. A distinctive feature of VR interventions is the requirement for active participation, which differentiates them from passive observation or therapist-guided movement.

Unlike traditional physiotherapy methods that often involve passive execution of movement, VR tasks require cognitive engagement and decision-making, such as initiating actions, planning movements and correcting errors. These processes activate neural pathways related to motor planning (prefrontal cortex, basal ganglia) and may support the development of both motor and cognitive functions. Such mechanisms are consistent with the concept of interactive learning [37,38,39]. Research has indicated that this form of interactive learning may be more effective in children, including those with neurological impairments, compared with methods that rely primarily on verbal instruction or observation.

One topic frequently addressed in the literature concerns the distinction between immersive and non-immersive VR systems. Immersive systems (e.g., VR goggles) fully immerse the user in a virtual environment, eliminating external stimuli and creating a strong sense of presence. By contrast, non-immersive systems (e.g., screen combined with video camera) allow interaction but without complete isolation from the external environment. Findings suggest that immersion may enhance a child’s engagement and improve the quality of movements [40]; however, in some children—particularly those with balance difficulties or sensory hypersensitivity—immersive systems may lead to discomfort, overload or anxiety. For this reason, the choice of system should be individualized and adjusted to the child’s specific needs and characteristics.

Another issue highlighted in the evaluation of VR effectiveness is the so-called novelty effect, referring to a temporary increase in motivation and performance caused by the excitement of using a new technology. This effect can complicate the assessment of whether therapeutic benefits are sustained over time. To address this limitation, future research should incorporate follow-up assessments, ideally at three- and six-months post-intervention, to better determine the durability and functional significance of VR outcomes [41].

This review also points to considerable heterogeneity in the assessment tools employed. While instruments such as AHA, QUEST, MA2 and PEDI-CAT are widely used and demonstrate good psychometric properties, there is currently no consensus on standardized outcome measures specifically for VR therapy. The absence of such standardization not only makes it difficult to compare findings across studies but also restricts the feasibility of quantitative meta-analyses. Establishing consensus on both outcome measures and minimum methodological requirements, therefore, appears to be an important next step in advancing this area of research.

When considering the use of VR in physiotherapy for children with cerebral palsy, it is also necessary to take into account the broader healthcare and organizational context. One of the main challenges in the management of pediatric neurological disorders is ensuring both continuity and accessibility of therapy. In many countries, access to specialized rehabilitation centers is limited, particularly outside major urban areas [42].

In this context, virtual reality may represent a potential tool to support therapy in home, community or remote settings. The introduction of VR into clinical practice has been associated with several logistical advantages. One frequently mentioned benefit is the possibility of providing more intensive therapy without proportionally increasing the number of therapists. After appropriate training of parents or legal guardians, children may be able to perform exercises independently or semi-independently, which could allow for an increased frequency of therapy sessions. In addition, monitoring technologies enable the collection of quantitative data that can be used to evaluate patient progress in the absence of direct therapist supervision. Systems such as those described by Choi et al. (2023) [16] include functions for remote progress monitoring, illustrating potential applications within telemedicine and telerehabilitation models.

The effects of VR therapy may also extend beyond motor outcomes. Children, especially at preschool and school age, participate in therapy within a wider social context that includes family, peers and school environments.

Home-based physiotherapy may help reduce the stress associated with commuting and scheduling therapy, while also creating opportunities for family members to be directly involved in the child’s rehabilitation process. Such involvement has been suggested to foster positive relationships and a sense of shared responsibility. Some evidence further indicates that the presence of a parent during VR therapy may enhance the child’s sense of safety and motivation to engage in exercises [43,44].

At the same time, potential challenges linked to the use of VR in children must be taken into account. Concerns have been raised about neurological and sensory safety, as certain children with cerebral palsy present with visual hypersensitivity or sensory processing difficulties that may be exacerbated by intensive VR stimuli. In such cases, inappropriate system selection could contribute to adverse effects including fatigue, dizziness, spatial disorientation, sensory overload, or, in rare cases, epileptic seizures in predisposed individuals [45]. Therefore, VR systems should be adapted to the individual characteristics and needs of each child, taking into consideration neurological, sensory and neuropsychological recommendations.

Based on the findings of this review and the available literature, the integration of VR with other technologies supporting neurological rehabilitation has been proposed as a promising direction for further research. For example, VR can be combined with functional robotics (such as hand exoskeletons) to provide simultaneous visual stimulation and facilitate actual motor execution. Studies investigating this type of combined intervention, often referred to as mixed reality rehabilitation, have suggested that it may enhance treatment effectiveness in children with more severe motor impairments who are unable to perform complete movement sequences independently [46].

Another potential role of VR concerns secondary prevention and the mitigation of disability progression. Some children with cerebral palsy, particularly those with milder functional limitations (GMFCS I–III), may restrict the spontaneous use of the affected limb, a phenomenon described as learned non-use. Due to its interactive and engaging design, VR has been suggested as a tool that may counteract this process by encouraging active use of the impaired limb within a motivating exercise environment.

When evaluating the outcomes of VR therapy, it is important to consider not only biomechanical parameters or standardized test scores, but also broader aspects such as quality of life for both the child and the family. For children who experience frustration due to limited manual dexterity, VR interventions may provide opportunities to build confidence and a sense of control. Participating in activities that are both therapeutic and enjoyable may help reduce the traditional divide between treatment and play. For parents, this could mean fewer conflicts during daily routines and greater independence for the child, which, in turn, may positively influence family well-being [47,48,49].

Despite increasing interest and a growing body of evidence, further clarification of optimal VR therapy protocols is still required. Large, multicenter randomized controlled trials with clearly defined protocols and long-term follow-up are necessary to assess not only the direct therapeutic effects of VR, but also its broader impact on quality of life, educational attainment and psychosocial development in children with cerebral palsy.

## 5. Conclusions

Based on the systematic review, several conclusions of significant importance for clinical practice and further research into physiotherapy for children with cerebral palsy (CP) can be drawn. The identified studies clearly indicate that virtual reality (VR), especially task-oriented therapy, immersive VR and gamified protocols, can effectively support the improvement of upper limb function in this patient population.

1. Virtual reality has a positive effect on neuroplasticity in children with cerebral palsy by activating multi-level brain pathways responsible for movement planning and control.
2. VR therapy can be a safe and effective tool to support traditional physiotherapy approaches in children with cerebral palsy, particularly in improving muscle strength, range of motion, coordination and grip.
3. Virtual reality is beneficial for patients with mild to moderate impairments (GMFCS I–III), while patients with more severe impairments require integration with assistive technologies (exoskeletons, robotics).
4. Remote therapy using VR can be a valuable alternative for families with limited access to specialist care.
5. Both immersive and non-immersive systems have therapeutic potential, but their selection should be tailored to the individual sensory and motor profile of the child.
6. Large-scale randomized clinical trials with long-term follow-up and clearly defined treatment protocols are necessary.
7. It is recommended to use standardized tools for assessing upper limb function (AHA, MA2, QUEST) and to analyze secondary effects (PEDI-CAT, quality of life).
8. There is evidence of the transfer of skills developed in a virtual reality environment to the activities of daily living. However, this evidence is inconclusive and requires confirmation in further scientific research.
9. The mechanisms of virtual reality effectiveness include: active patient participation, an engaging multisensory environment, increased repetition and intensification of exercises.

## Figures and Tables

**Table 1:** Results of VR therapy in children with cerebral palsy.

	Authors (Year)	Study Type	Number of Participants	Age	CP Type	VR	Therapy Duration
1	Menekseoglu et al. (2023) [15]	RCT	36	6–12 years	hemiplegia	gamified VR	6 weeks (3×/week)
2	Choi et al. (2023) [16]	RCT	40	4–17 years	hemiplegia, diplegia	VR + IMU	4 weeks (daily)
3	Goyal et al. (2022) [17]	RCT	10	6–12 years	hemiplegia	VR + IMU	6 weeks (daily)
4	Metin Ökmen et al. (2019) [18]	RCT	41	5–15 years	hemiplegia	task-oriented VR	4 weeks (3×/week)
5	Daliri et al. (2025) [19]	RCT	30	6–10 years	hemiplegia	task-oriented VR	8 weeks (2×/week)
6	Saussez et al. (2023) [20]	RCT	40	5–18 years	hemiplegia	task-oriented VR	8 weeks (2×/week)
7	Baillet et al. (2024) [21]	RCT	20	6–12 years	hemiplegia	immersive 3D VR	5 weeks (3×/week)
8	Avcil et al. (2021) [22]	RCT	30	8–12 years	hemiplegia	leap Motion	6 weeks
9	Golomb et al. (2010) [23]	RCT—in-home VR	30	13–17 years	hemiplegia	VR + telerehabilitation	6 weeks

## Data Availability

The data presented in this study are available on request from the corresponding author.
